# Crystal structures and electrochemical properties of nickel(II) complexes with *N*,*N*′,*N*′′,*S*-tetra­dentate Schiff base ligands

**DOI:** 10.1107/S2056989022003954

**Published:** 2022-04-22

**Authors:** Masakazu Hirotsu, Junhei Sanou, Toyotaka Nakae, Takumi Matsunaga, Isamu Kinoshita

**Affiliations:** a Department of Chemistry, Faculty of Science, Kanagawa University, 2946 Tsuchiya, Hiratsuka, Kanagawa, 259-1293, Japan; b Graduate School of Science, Osaka City University, Sumiyoshi-ku, Osaka 558-8585, Japan

**Keywords:** crystal structure, nickel(II) complex, thiol­ate, tetra­dentate Schiff base ligand, piperazine, proton reduction

## Abstract

The thiol­ate nickel complexes {2-[({2-[(2-amino­ethyl-κ*N*)(meth­yl)amino-κ*N*]eth­yl}imino-κ*N*)meth­yl]benzene­thiol­ato-κ*S*}nickel(II) chloride, [Ni(C_12_H_18_N_3_S)]Cl (**1**), and [2-({[2-(piperazin-1-yl-κ^2^ *N* ^1^,*N* ^4^)eth­yl]imino-κ*N*}meth­yl)benzene­thiol­ato-κ*S*]nickel(II) hexa­fluoro­phosphate di­chloro­methane monosolvate, [Ni(C_13_H_18_N_3_S)]PF_6_·CH_2_Cl_2_ (**2**), were synthesized by the reactions of 2-(*tert*-butyl­thio)­benzaldehyde, tri­amines, and nickel(II) salts. Both complexes have a nickel ion surrounded by an *N*,*N*′,*N*′′,*S*-tetra­dentate ligand, forming a square-planar geometry. The terminal *N*,*N*-chelating moiety is *N*,*N*-di­alkyl­ethane-1,2-di­amine for **1** and 1-alkyl­piperazine for **2**. The N—Ni—N bite angle in the terminal *N*,*N*-chelate ring in **2** [76.05 (10)°] is much smaller than that in **1** [86.16 (6)°]. Cyclic voltammograms of **1** and **2** in aqueous media indicated that the reduction and oxidation potentials of **2** are more positive than those of **1**. The smaller bite angle of the terminal piperazine chelate in **2** reduces the electron-donating ability of the tetra­dentate ligand, resulting in a positive shift of the redox potentials. Both complexes exhibit catalytic activity for proton reduction, and the piperazine moiety in **2** is effective in reducing the overpotential.

## Abstract 2

Nickel(II) Schiff base complexes containing thiol­ate S and polyamine N donor atoms exhibit electrocatalytic activity for proton reduction. The piperazine moiety in the Schiff base ligand gives a smaller bite angle, which is effective in reducing the overpotential.

## 1. Chemical context

Sulfur donor atoms bound to iron or nickel ions are commonly found in the active site of hydrogenase enzymes in nature. In [NiFe] hydrogenases, cysteine sulfurs are bound to the metal centers, and in [FeFe] hydrogenases, the amine moiety in the aza­dithiol­ate ligand bound to the iron centers is essential to the catalytic function (Lubitz *et al.*, 2014 ▸). Nickel(II) complexes with sulfur and nitro­gen donor atoms are efficient precatalysts or real catalysts for the electro- and photoreduction of protons (Han *et al.*, 2012 ▸; Martin *et al.*, 2015 ▸; Luo *et al.*, 2017 ▸; Inoue *et al.*, 2020 ▸). It has been pointed out that the hemilabile pyridine ligand in [Ni(C_5_H_4_NS)_3_] is protonated in the photocatalytic hydrogen production (Han *et al.*, 2012 ▸). The pendant amines as a proton acceptor site are also important for developing efficient electrocatalysts for hydrogen production (Helm *et al.*, 2011 ▸; Stewart *et al.*, 2013 ▸). In this context, thiol­ate complexes with pendant amino groups are good candidates for the development of proton-reduction catalysts.

The nickel(II) complex [Ni(C_11_H_16_N_3_S)]Cl (Bouwman *et al.*, 1999 ▸) contains an *N*,*N*′,*N*′′,*S*-tetra­dentate Schiff base ligand with terminal thiol­ate and amine moieties. The terminal amino group that is bound to the Ni center is a potential proton-acceptor site. For instance, the Schiff base ligands derived from salicyl­aldehydes and 1-(2-amino­eth­yl)piperazine give square-planar and/or octa­hedral nickel(II) complexes, in which the terminal piperazinyl group binds to Ni in the bidentate chelate mode and the monodentate mode with protonation (Mukhopadhyay *et al.*, 2003 ▸). Furthermore the cationic complex [Ni(C_11_H_16_N_3_S)]Cl is water-soluble, which makes it possible to investigate its catalytic performance in aqueous media. In the electrocatalytic proton reduction, the electrochemical properties of the precatalysts are directly related to the formation of real catalysts. Therefore the tuning of the redox properties is also required in the ligand design. 
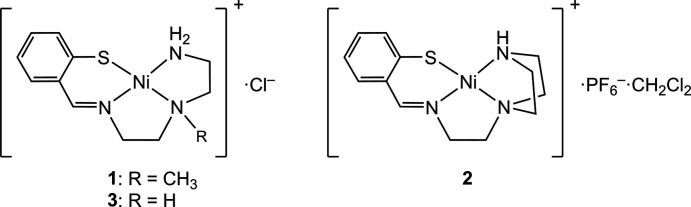


In this work we synthesized two water-soluble N_3_S Schiff base nickel(II) complexes, [Ni(C_12_H_18_N_3_S)]Cl (**1**) and [Ni(C_13_H_18_N_3_S)]PF_6_ (**2**), in which the *N*,*N*′,*N*′′,*S*-tetra­dentate Schiff base ligands contain an additional *N*-methyl group and a terminal piperazine moiety, respectively. The electrochemical properties of these complexes were investigated by cyclic voltammetry in water, and compared with those of [Ni(C_11_H_16_N_3_S)]Cl (**3**) without N-substituents.

## 2. Structural commentary

The complex cations in **1** and **2** consist of an Ni^2+^ ion and a monoanionic *N*,*N*′,*N*′′,*S*-tetra­dentate ligand, giving a square-planar geometry. The asymmetric unit in **1** comprises the complex cation and a chloride anion, whereas in **2** a hexa­fluoro­phosphate anion and a di­chloro­methane mol­ecule are incorporated into the crystal lattice.

**Figure 1:**
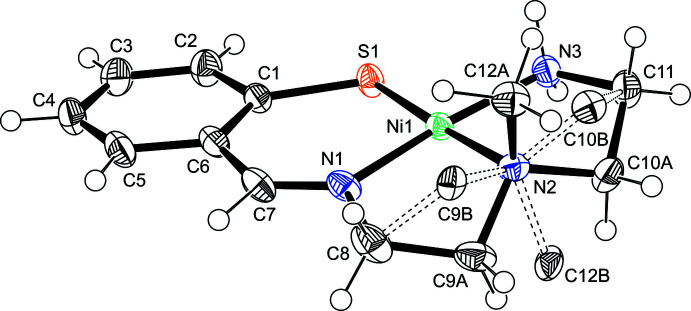
Perspective view of the complex cation of **1** with displacement ellipsoids at the 50% probability level. Hydrogen atoms of the minor occupancy component of the disordered region are omitted for clarity.

**Figure 2:**
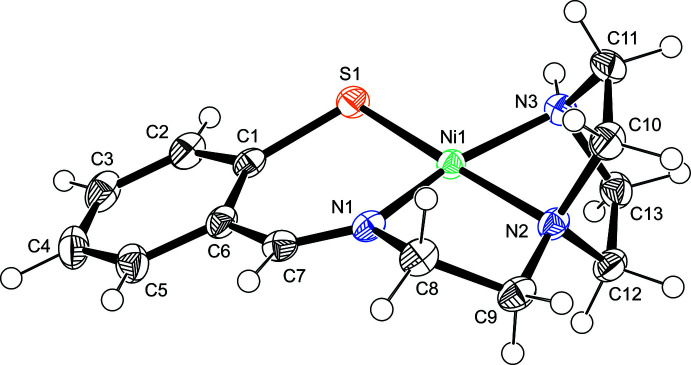
Perspective view of the complex cation of **2** with displacement ellipsoids at the 50% probability level.

Each complex cation contains a six-membered chelate ring with N and S donor atoms and two five-membered chelate rings with two N donor atoms (Fig. 1 ▸ and Fig. 2 ▸). In the *N,S*-chelate, the N1—Ni1—S1 angles are 97.77 (5)° in **1** (Table 1 ▸) and 98.90 (7)° in **2** (Table 2 ▸), which are comparable to those in **3** [98.5 (2)°] and the tetra­phenyl­borate salt [Ni(C_11_H_16_N_3_S)]B(C_6_H_5_)_4_ [**3′**; 95.8 (2)°]. The bond distances in the chelate rings are also comparable (Bouwman *et al.*, 1999 ▸; Goswami & Eichhorn, 1999 ▸). The structural parameters of the central five-membered *N*,*N*-chelate rings of these complexes are similar to each other: the N1—Ni1—N2 angles are 86.53 (6)° in **1**, 87.80 (10)° in **2**, 86.1 (3)° in **3** (Bouwman *et al.*, 1999 ▸), and 87.8 (3)° in **3′** (Goswami & Eichhorn, 1999 ▸). The bite angles of the *N*,*N*-chelate are also similar to those of the nickel(II) complexes with *S,N,N,S*-tetra­dentate ligands, which have two amine N or two imine N donor atoms besides two S atoms (Yamamura *et al.*, 1993 ▸). The structures of the central NCH_2_CH_2_N chelate are not significantly dependent on the terminal chelates of the tetra­dentate ligands. In the terminal *N*,*N*-chelate ring, the bite angle of the piperazine moiety is significantly restricted. The N2—Ni1—N3 angle of **2** [76.05 (10)°] is much smaller than those of **1** [86.16 (6)°], **3** [86.3 (3)°; Bouwman *et al.*, 1999 ▸], and **3′** [86.9 (3)°; Goswami & Eichhorn, 1999 ▸], although the Ni—N distances match well with each other.

In complex **1**, the methyl­ene chains in the two *N*,*N*-chelate rings and the methyl group on the tertiary amine N atom are disordered over two sets of sites. These two models are enanti­omers to each other. The conformation of the methyl­ene chains is dependent on the configuration of the methyl group. The *N,S*-chelate ring in **1** does not show disorder, and the benzene ring and the NiN_3_S coordination plane are almost coplanar. The dihedral angle between the least-squares planes is 9.74 (8)°. The corresponding inter­planar angle in **2** is 20.92 (12)°. Because there is no significant difference in the conformation of the central chelate ring between **1** [N1—C8—C9*A*—N2 = 42.9 (2)°] and **2** [N1—C8—C9—N2 = 45.1 (3)°], this bending is due to the rigid structure of the piperazine chelate, which fixes the direction of the methyl­ene groups on the tertiary amine N atom.

## 3. Supra­molecular features

**Figure 3:**
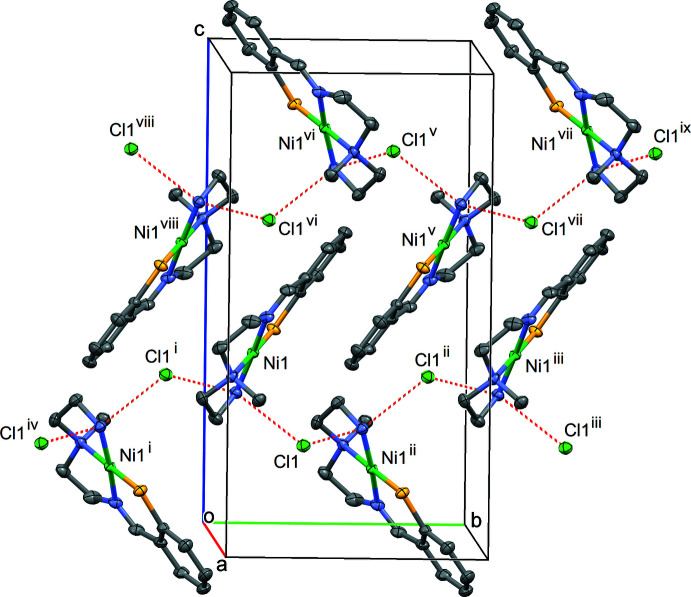
Hydrogen-bond network of **1**. Hydrogen atoms are omitted for clarity. Hydrogen bonds are shown as red dashed lines. [Symmetry codes: (i) −*x* + 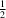 , *y* − 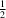 , −*z* + 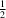 ; (ii) −*x* + 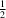 , *y* + 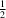 , −*z* + 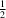 ; (iii) *x*, *y* + 1, *z*; (iv) *x*, *y* − 1, *z*; (v) −*x* + 1, −*y* + 1, −*z* + 1; (vi) *x* + 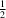 , −*y* + 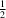 , *z* + 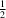 ; (vii) *x* + 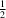 , −*y* + 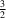 , *z* + 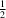 ; (viii) −*x* + 1, −*y*, −*z* + 1; (ix) −*x* + 1, −*y* + 2, −*z* + 1.]

The crystal structure of **1** shows hydrogen bonds between the terminal amine nitro­gen atom in the complex cation and two chloride ions with the N3⋯Cl1 and N3⋯Cl1(−*x* + 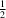 , *y* − 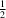 , −*z* + 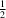 ) distances of 3.2245 (17) and 3.1948 (17) Å, respectively (Table 3 ▸), which are similar to that of **3** (Bouwman *et al.*, 1999 ▸). Each chloride ion bridges two complex cations through the hydrogen bonds. Thus in the crystal, the cations and anions pack together to form a zigzag hydrogen-bonded chain along the *b*-axis direction (Fig. 3 ▸). The disorder found in the complex cation does not affect the chain structure. There are π–π inter­actions between the hydrogen-bonded chains through the planar *N,S*-chelate moieties including the benzene rings [centroid–centroid distances = 3.7378 (12) and 3.8965 (13) Å].

Several inter­molecular C—H⋯π inter­actions exist in **2** between the methyl­ene hydrogen atoms of the polyamine moiety and the π system of the benzene ring (Table 4 ▸). The piperazine nitro­gen atom N3 in the ligand forms a hydrogen bond to the hexa­fluoro­phosphate ion with the N3⋯F6(−*x* + 1, −*y* + 1, −*z* + 1) distance of 3.114 (3) Å (Table 4 ▸). In addition, there are short contacts between the hexa­fluoro­phosphate ion and the methyl­ene hydrogen atoms of the ligand [F6⋯H14*B*(*x*, *y*, *z* − 1) = 2.47 (4) Å].

## 4. Database survey

The two *N*,*N*′,*N*′′,*S*-tetra­dentate Schiff base ligands studied here have not been reported so far for other transition-metal ions. A similar Schiff base structure that contains benzene­thiol­ate and polyamines is found in a trinuclear nickel(II) complex with a *C*_3_-symmetric ligand based on a 1,3,5-trimercapto­benzene backbone (Feldscher *et al.*, 2014 ▸). An analogous mononuclear nickel(II) complex that has a phenol O atom instead of the thiol S atom in **2** shows a piperazine bite angle of 76.65 (8)° (Mukhopadhyay *et al.*, 2003 ▸), which is comparable to that of **2**.

## 5. Spectroscopic features

**Figure 4:**
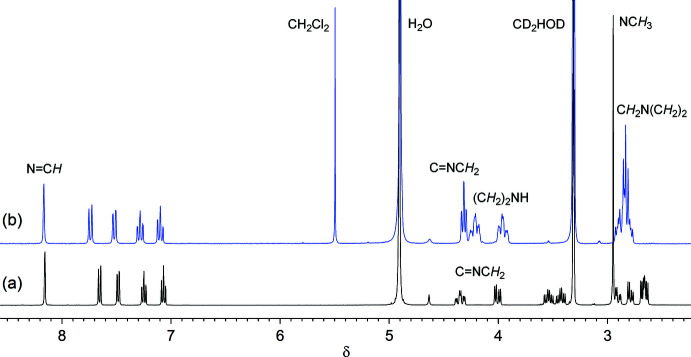
^1^H NMR spectra of (*a*) **1** (400 MHz) and (*b*) **2** (300 MHz) in CD_3_OD.

The solution structures of **1** and **2** were characterized by ^1^H and ^1^H–^1^H COSY NMR spectroscopy in methanol-*d*_4_. The ^1^H NMR spectrum of **1** exhibits an azomethine proton at 8.16 ppm and four aromatic protons in the range 7.07–7.65 ppm (Fig. 4 ▸). In the aliphatic region, eight multiplet signals and a singlet signal are due to methyl­ene and methyl groups, respectively. The COSY spectrum of **1** shows cross peaks between the azomethine proton and the two methyl­ene protons at 4.01 and 4.35 ppm; thus, they were attributed to the CH_2_ group adjacent to the C=N group. Similar spectroscopic features appear for **2** in the aromatic region, whereas six sets of signals due to methyl­ene protons are observed in the aliphatic region. The two sharp signals at 2.83 and 4.31 ppm for **2** were attributed to the central *N*,*N*-chelate moiety, and the latter is assigned to the CH_2_ group adjacent to C=N on the basis of the COSY correlation. This observation is consistent with the fast conformational change of the central chelate ring in **2**. Furthermore, the similar signal pattern for the terminal methyl­ene protons at 3.96 and 4.21 ppm suggests a boat conformation of the piperazine moiety that binds to Ni in the bidentate chelate mode.

## 6. Electrochemical Properties

**Figure 5:**
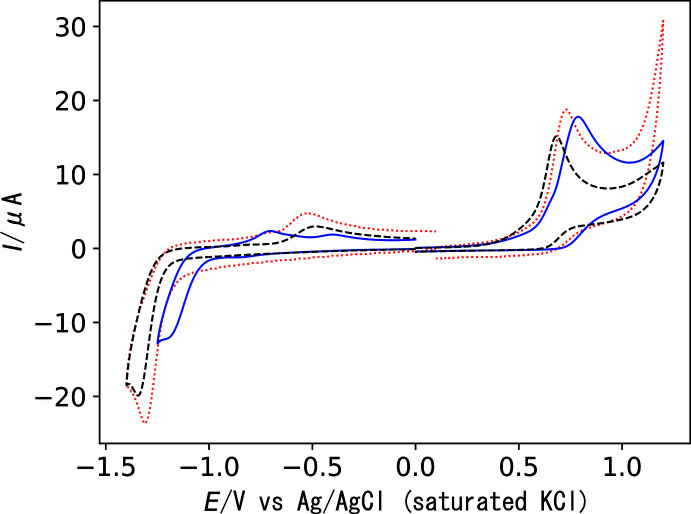
Cyclic voltammograms of complexes **1** (0.5 m*M*, red dotted line), **2** (0.5 m*M*, blue solid line), and **3** (0.5 m*M*, black dashed line) in water containing 0.10 *M* KNO_3_: scan rate, 0.1 V s^−1^; working electrode, glassy carbon; auxiliary electrode, platinum wire; reference electrode, Ag/AgCl/saturated KCl.

The redox behavior of the *N*,*N*′,*N*′′,*S*-tetra­dentate Schiff base nickel(II) complexes **1**, **2**, and **3** was investigated by cyclic voltammetry. Measurements were performed in 5 × 10^−4^ *M* (1 *M* = 1 mol dm^−3^) aqueous solution containing KNO_3_ (0.1 *M*) at a scan rate of 0.1 V s^−1^. The working electrode was a glassy carbon disk electrode with a diameter of 3 mm, the auxiliary electrode was a platinum wire, and the reference electrode was Ag/AgCl/saturated KCl. All complexes exhibit irreversible reduction and oxidation processes (Fig. 5 ▸). In the reduction process, the cathodic wave appeared at −1.31 V for **1**, −1.19 V for **2**, and −1.34 V for **3**. The anodic peaks in the reverse scan (–0.52 V for **1**; −0.70, −0.40 V for **2**, and −0.48 V for **3**) suggest the adsorption of the reduced species. In the oxidation process, the anodic wave appeared at 0.73 V for **1**, 0.79 V for **2**, and 0.68 V for **3**. In both processes, the redox potentials of **1** are slightly shifted to more positive values than those of **3**, which suggests that the electronic and steric effects of the methyl group on the central N atom are not so significant. The voltammogram of **2** shows further positive shifts, and the shift in the reduction process is more pronounced. This is probably related to the smaller bite angle of the terminal piperazine chelate, which reduces the electron-donating ability of the Schiff base ligand toward the nickel center.

**Figure 6:**
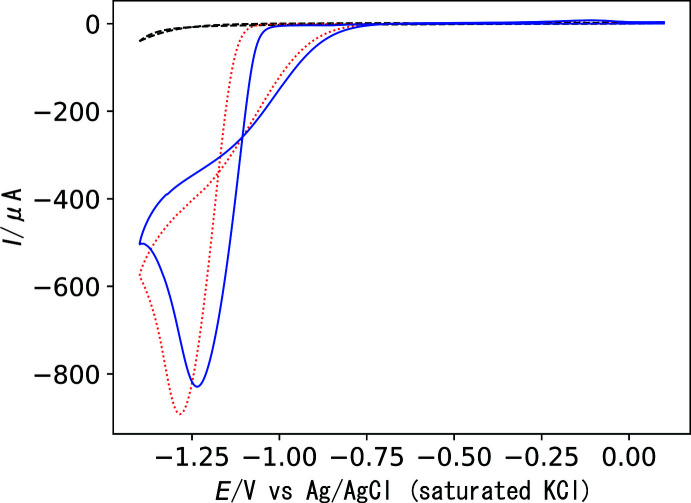
Cyclic voltammograms of complexes **1** (0.5 m*M*, red dotted line), **2** (0.5 m*M*, blue solid line), and blank solution (black dashed line) in 0.1 *M* acetate buffer at pH 4.6 containing 0.10 *M* KNO_3_: scan rate, 0.1 V s^−1^; working electrode, glassy carbon; auxiliary electrode, platinum wire; reference electrode, Ag/AgCl/saturated KCl.

The proton-reduction abilities of complexes **1** and **2** were compared in a buffer solution of pH 4.6 (0.1 *M* acetic acid/sodium acetate). A catalytic current was observed during the reduction process, giving a peak at −1.28 V for **1** and −1.23 V for **2** (Fig. 6 ▸). This suggests that the reduced species of the nickel(II) complex is catalytically active for proton reduction. The reduction potential for **2** is more positive than that for **1**, and thus the piperazinyl arm in **2** is effective in reducing the overpotential for proton reduction.

## 7. Synthesis and crystallization

**General Procedures.** NMR spectra were recorded on a Bruker AVANCE 300 or a JEOL EX-400 spectrometer at room temperature. Cyclic voltammetric measurements were performed at room temperature with an ALS/DY2325 voltammetric analyzer (Bioanalytical System Ins.) under N_2_. Elemental analyses were performed by the Analytical Research Service Center at Osaka City University or A Rabbit Science Co., Ltd.

**[Ni(C_12_H_18_N_3_S)]Cl (1).** A solution of 2,2′-di­amino-*N*-methyl­diethyl­amine (128 µL, 1.0 mmol) and 2-(*t*-butyl­thio)­benzaldehyde (194 mg, 1.0 mmol) in ethanol (10 mL) was refluxed under a nitro­gen atmosphere for 1 h to afford a pale-yellow solution. After cooling to room temperature, NiCl_2_·6H_2_O (238 mg, 1.0 mmol) was added, and the resulting green suspension was refluxed under a nitro­gen atmosphere for 6 h, during which time the color of the solution turned red. The reaction mixture was cooled to room temperature and filtered. The red filtrate was left for two weeks to give red crystals of **1** (41 mg, 12%). ^1^H NMR (400 MHz, CD_3_OD): δ 2.65 (*dd*, *J* = 12.1, 4.9 Hz, 1H, C=NCH_2_C*H*_2_N), 2.67 (*dd*, *J* = 12.2, 4.5 Hz, 1H, NC*H*_2_CH_2_NH_2_), 2.79 (*dd*, *J* = 13.3, 5.9 Hz, 1H, NCH_2_C*H*_2_NH_2_), 2.92 (*dd*, *J* = 13.3, 4.5 Hz, 1H, NCH_2_C*H*_2_NH_2_), 2.95 (*s*, 3H, N*Me*), 3.43 (*td*, *J* = 12.8, 5.9 Hz, 1H, NC*H*_2_CH_2_NH_2_), 3.54 (*td*, *J* = 12.8, 6.1 Hz, 1H, C=NCH_2_C*H*_2_N), 4.01 (*dd*, *J* = 15.1, 6.1 Hz, 1H, C=NC*H*_2_CH_2_N), 4.35 (*dddd*, *J* = 15.1, 13.5, 4.9, 1.7 Hz, 1H, C=NC*H*_2_CH_2_N), 7.07 (*ddd*, *J* = 7.8, 7.2, 1.1 Hz, 1H, Ar) 7.25 (*ddd*, *J* = 8.0, 7.2, 1.4 Hz 1H, Ar), 7.49 (*dd*, *J* = 8.0, 1.3 Hz, 1H, Ar), 7.65 (*d*, *J* = 8.1 Hz, 1H, Ar), 8.16 (*s*, 1H, N=C*H*). Analysis calculated for C_12_H_18_ClN_3_NiS·0.25H_2_O: C, 43.02; H, 5.57; N, 12.54. Found: C, 42.94; H, 5.28; N, 12.48.

**[Ni(C_13_H_18_N_3_S)]PF_6_ (2).** To a solution of *N*-(2-amino­eth­yl)piperazine (261 mg, 2.0 mmol) and 2-(*t*-butyl­thio)­benzaldehyde (490 mg, 2.1 mmol) in ethanol (20 mL) was added NiCl_2_·6H_2_O (490 mg, 2.1 mmol). The resulting suspension was refluxed under a nitro­gen atmosphere for 8 h, during which time the color of the solution turned orange and a yellow–green precipitate formed. The reaction mixture was filtered, and an ethanol solution (5 mL) of ammonium hexa­fluoro­phosphate (334 mg, 2.1 mmol) was added to the filtrate. The resulting orange precipitate was collected by filtration and dried under reduced pressure to give an orange powder of **2** (353 mg, 38%). Suitable crystals for X-ray diffraction analysis were grown from a di­chloro­methane solution by layering with diethyl ether. ^1^H NMR (300 MHz, CD_3_OD): δ 2.76–2.94 (*m*, 4H, N(C*H*_2_CH_2_)_2_NH), 2.83 (*t*, *J* = 6.5 Hz, 2H, C=NCH_2_C*H*_2_N), 3.91–4.03 (*m*, 2H, N(CH_2_C*H*_2_)_2_NH), 4.16–4.27 (*m*, 2H, N(CH_2_C*H*_2_)_2_NH), 4.31 (*td*, *J* = 6.5, 1.2 Hz, 2H, C=NC*H*_2_CH_2_N), 7.10 (*td*, *J* = 7.9, 1.2 Hz, 1H, Ar), 7.28 (*td*, *J* = 8.2, 1.5 Hz, 1H, Ar), 7.52 (*dd*, *J* = 7.9, 1.5 Hz, 1H, Ar), 7.74 (*d*, *J* = 8.2 Hz, 1H, Ar), 8.16 (*s*, 1H, N=C*H*). Analysis calculated for C_13_H_18_F_6_N_3_NiPS·0.75CH_2_Cl_2_: C, 32.02; H, 3.81; N, 8.15. Found: C, 32.11; H, 3.93; N, 8.14.

## 8. Refinement

Crystal data, data collection and structure refinement details are summarized in Table 5 ▸. All non-hydrogen atoms were refined anisotropically. A methyl group and two methyl­ene groups bound to the central N atom of two *N*,*N*-chelating moieties in **1** were modeled as disordered over two positions each, and the occupancy factors refined to 0.864 (3) and 0.136 (3). Hydrogen atoms on the disordered C atoms and the adjacent C atoms that belong to the minor site were placed in calculated positions with C—H(meth­yl) = 0.98 Å and C—H(methyl­ene) = 0.99 Å and refined using a riding model with *U*_iso_(H) = 1.5*U*_eq_(C) and 1.2*U*_eq_(C), respectively. Other H atoms were found in a difference-Fourier map and freely refined.

## Supplementary Material

Crystal structure: contains datablock(s) 1, 2. DOI: 10.1107/S2056989022003954/dj2045sup1.cif

Structure factors: contains datablock(s) 1. DOI: 10.1107/S2056989022003954/dj20451sup2.hkl

Structure factors: contains datablock(s) 2. DOI: 10.1107/S2056989022003954/dj20452sup3.hkl

CCDC references: 2165891, 2165890

Additional supporting information:  crystallographic information; 3D view; checkCIF report

## Figures and Tables

**Table 1:** Selected geometric parameters (Å, °) for **1** [Chem scheme1]

Ni1—N1	1.8587 (14)	Ni1—S1	2.1421 (5)
Ni1—N3	1.9268 (14)	S1—C1	1.7396 (17)
Ni1—N2	1.9345 (13)	N1—C7	1.289 (2)
			
N1—Ni1—N3	172.68 (6)	N1—Ni1—S1	97.77 (5)
N1—Ni1—N2	86.53 (6)	N3—Ni1—S1	89.53 (5)
N3—Ni1—N2	86.16 (6)	N2—Ni1—S1	175.49 (4)
			
N1—C8—C9*A*—N2	42.9 (2)		

**Table 2:** Selected geometric parameters (Å, °) for **2** [Chem scheme1]

Ni1—N1	1.843 (2)	Ni1—S1	2.1316 (10)
Ni1—N3	1.917 (3)	S1—C1	1.745 (3)
Ni1—N2	1.924 (2)	N1—C7	1.285 (3)
			
N1—Ni1—N3	162.95 (10)	N1—Ni1—S1	98.90 (7)
N1—Ni1—N2	87.80 (10)	N3—Ni1—S1	97.67 (8)
N3—Ni1—N2	76.05 (10)	N2—Ni1—S1	171.77 (7)
			
N1—C8—C9—N2	45.1 (3)		

**Table 3:** Hydrogen-bond geometry (Å, °) for **1** [Chem scheme1]

*D*—H⋯*A*	*D*—H	H⋯*A*	*D*⋯*A*	*D*—H⋯*A*
N3—H*N*3*A*⋯Cl1	0.93 (2)	2.40 (2)	3.2245 (17)	148.7 (18)
N3—H*N*3*B*⋯Cl1^i^	0.841 (19)	2.41 (2)	3.1948 (17)	156.0 (17)

Symmetry code: (i) 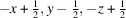 .

**Table 4:** Hydrogen-bond geometry (Å, °) for **2** [Chem scheme1] *Cg*6 is the centroid of the C1–C6 ring.

*D*—H⋯*A*	*D*—H	H⋯*A*	*D*⋯*A*	*D*—H⋯*A*
N3—H3*N*⋯F6^i^	0.80 (3)	2.50 (3)	3.114 (3)	135 (3)
C10—H10*A*⋯C4^ii^	0.96 (3)	2.85 (3)	3..695 (5)	147 (2)
C8—H8*A*⋯*Cg*6^ii^	0.98 (3)	2.84 (3)	3.778 (4)	160 (2)

Symmetry codes: (i) 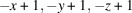 ; (ii) 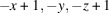 .

**Table 5:** Experimental details

	**1**	**2**
Crystal data		
Chemical formula	[Ni(C_12_H_18_N_3_S)]Cl	[Ni(C_13_H_18_N_3_S)]PF_6_·CH_2_Cl_2_
*M*_r_	330.51	536.97
Crystal system, space group	Monoclinic, *P*2_1_/*n*	Triclinic, *P* 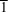
Temperature (K)	153	153
*a*, *b*, *c* (Å)	7.8601 (14), 9.8884 (17), 18.190 (3)	8.725 (3), 10.507 (4), 11.316 (4)
α, β, γ (°)	90, 98.677 (3), 90	98.065 (4), 101.274 (6), 96.150 (5)
*V* (Å^3^)	1397.6 (4)	997.6 (6)
*Z*	4	2
Radiation type	Mo *K*α	Mo *K*α
μ (mm^−1^)	1.71	1.49
Crystal size (mm)	0.11 × 0.06 × 0.04	0.17 × 0.11 × 0.08

Data collection		
Diffractometer	Rigaku AFC11 with Saturn 724+ CCD	Rigaku AFC11 with Saturn 724+ CCD
Absorption correction	Multi-scan (*REQAB*; Rigaku, 1998 ▸)	Multi-scan (*REQAB*; Rigaku, 1998 ▸)
*T*_min_, *T*_max_	0.904, 1.000	0.828, 1.000
No. of measured, independent and observed [*I* > 2σ(*I*)] reflections	11199, 3129, 2665	8155, 4392, 3240
*R*_int_	0.024	0.041
(sin θ/λ)_max_ (Å^−1^)	0.649	0.649

Refinement		
*R*[*F* ^2^ > 2σ(*F* ^2^)], *wR*(*F* ^2^), *S*	0.024, 0.057, 1.05	0.037, 0.087, 0.93
No. of reflections	3129	4392
No. of parameters	263	333
H-atom treatment	H atoms treated by a mixture of independent and constrained refinement	All H-atom parameters refined
Δρ_max_, Δρ_min_ (e Å^−3^)	0.43, −0.24	0.79, −0.52

Computer programs: *CrystalClear* (Rigaku, 2008 ▸), *SIR97* (Altomare *et al.*, 1999 ▸), *SHELXL2013* (Sheldrick, 2015 ▸), *ORTEP-3 for Windows* and *WinGX* (Farrugia, 2012 ▸), and *Mercury* (Macrae *et al.*, 2020 ▸).

## supplementary crystallographic information

### {2-[({2-[(2-Aminoethyl-κN)(methyl)amino-κN]ethyl}imino-κN)methyl]benzenethiolato-κS}nickel(II) chloride (1) Crystal data

**Table:**

[Ni(C_12_H_18_N_3_S)]Cl	*F*(000) = 688
*M**_r_* = 330.51	*D*_x_ = 1.571 Mg m^−^^3^
Monoclinic, *P*2_1_/*n*	Mo *K*α radiation, λ = 0.71075 Å
*a* = 7.8601 (14) Å	Cell parameters from 4130 reflections
*b* = 9.8884 (17) Å	θ = 3.1–27.5°
*c* = 18.190 (3) Å	µ = 1.71 mm^−^^1^
β = 98.677 (3)°	*T* = 153 K
*V* = 1397.6 (4) Å^3^	Prism, red
*Z* = 4	0.11 × 0.06 × 0.04 mm

### {2-[({2-[(2-Aminoethyl-κN)(methyl)amino-κN]ethyl}imino-κN)methyl]benzenethiolato-κS}nickel(II) chloride (1) Data collection

**Table:**

Rigaku AFC11 with Saturn 724+ CCD diffractometer	3129 independent reflections
Radiation source: Rotating Anode	2665 reflections with *I* > 2σ(*I*)
Detector resolution: 28.5714 pixels mm^-1^	*R*_int_ = 0.024
ω scans	θ_max_ = 27.5°, θ_min_ = 3.1°
Absorption correction: multi-scan (*REQAB*; Rigaku, 1998)	*h* = −10→10
*T*_min_ = 0.904, *T*_max_ = 1.000	*k* = −12→11
11199 measured reflections	*l* = −23→22

### {2-[({2-[(2-Aminoethyl-κN)(methyl)amino-κN]ethyl}imino-κN)methyl]benzenethiolato-κS}nickel(II) chloride (1) Refinement

**Table:**

Refinement on *F*^2^	Primary atom site location: structure-invariant direct methods
Least-squares matrix: full	Secondary atom site location: difference Fourier map
*R*[*F*^2^ > 2σ(*F*^2^)] = 0.024	Hydrogen site location: mixed
*wR*(*F*^2^) = 0.057	H atoms treated by a mixture of independent and constrained refinement
*S* = 1.05	*w* = 1/[σ^2^(*F*_o_^2^) + (0.0319*P*)^2^ + 0.1838*P*] where *P* = (*F*_o_^2^ + 2*F*_c_^2^)/3
3129 reflections	(Δ/σ)_max_ = 0.001
263 parameters	Δρ_max_ = 0.43 e Å^−^^3^
0 restraints	Δρ_min_ = −0.24 e Å^−^^3^

### {2-[({2-[(2-Aminoethyl-κN)(methyl)amino-κN]ethyl}imino-κN)methyl]benzenethiolato-κS}nickel(II) chloride (1) Special details

**Table:**

Geometry. All esds (except the esd in the dihedral angle between two l.s. planes) are estimated using the full covariance matrix. The cell esds are taken into account individually in the estimation of esds in distances, angles and torsion angles; correlations between esds in cell parameters are only used when they are defined by crystal symmetry. An approximate (isotropic) treatment of cell esds is used for estimating esds involving l.s. planes.
Refinement. A methyl group and two methylene groups bound to N2 were modeled as disordered over two positions each, and the occupancy factors were refined to 0.864 (3) and 0.136 (3).

### {2-[({2-[(2-Aminoethyl-κN)(methyl)amino-κN]ethyl}imino-κN)methyl]benzenethiolato-κS}nickel(II) chloride (1) Fractional atomic coordinates and isotropic or equivalent isotropic displacement parameters (Å^2^)

**Table:**

	*x*	*y*	*z*	*U*_iso_*/*U*_eq_	Occ. (<1)
Ni1	0.48400 (3)	0.13970 (2)	0.38534 (2)	0.01822 (7)	
Cl1	0.40893 (5)	0.34256 (4)	0.18937 (2)	0.02646 (10)	
S1	0.26456 (5)	0.23714 (4)	0.41873 (2)	0.02246 (10)	
N1	0.64016 (17)	0.17644 (15)	0.47031 (8)	0.0235 (3)	
N2	0.66981 (17)	0.04423 (14)	0.34932 (8)	0.0215 (3)	
N3	0.34538 (19)	0.08851 (16)	0.29300 (8)	0.0225 (3)	
C1	0.3229 (2)	0.33075 (16)	0.49958 (9)	0.0217 (3)	
C2	0.1926 (2)	0.4054 (2)	0.52601 (10)	0.0288 (4)	
C3	0.2272 (3)	0.4853 (2)	0.58865 (11)	0.0329 (4)	
C4	0.3922 (3)	0.4947 (2)	0.62816 (10)	0.0330 (4)	
C5	0.5199 (2)	0.4198 (2)	0.60486 (10)	0.0293 (4)	
C6	0.4898 (2)	0.33615 (17)	0.54095 (9)	0.0236 (4)	
C7	0.6332 (2)	0.25697 (19)	0.52548 (9)	0.0270 (4)	
C8	0.8076 (2)	0.1028 (2)	0.47227 (11)	0.0344 (5)	
C9A	0.7758 (3)	−0.0122 (2)	0.41595 (12)	0.0309 (5)	0.864 (3)
C9B	0.8355 (14)	0.1034 (12)	0.3869 (7)	0.019 (3)	0.136 (3)
C10A	0.5894 (3)	−0.0624 (2)	0.29769 (13)	0.0287 (5)	0.864 (3)
C11	0.4437 (2)	0.0035 (2)	0.24619 (10)	0.0279 (4)	
C10B	0.6424 (15)	0.0424 (13)	0.2701 (7)	0.022 (3)	0.136 (3)
C12A	0.7731 (3)	0.1392 (2)	0.30855 (12)	0.0277 (5)	0.864 (3)
C12B	0.6622 (19)	−0.1036 (12)	0.3796 (8)	0.026 (3)	0.136 (3)
HN3A	0.321 (3)	0.170 (2)	0.2682 (12)	0.041 (6)*	
HN3B	0.259 (2)	0.044 (2)	0.3013 (10)	0.025 (5)*	
H2	0.077 (2)	0.3969 (18)	0.5015 (10)	0.021 (5)*	
H3	0.140 (3)	0.530 (2)	0.6037 (11)	0.035 (6)*	
H4	0.415 (3)	0.551 (2)	0.6699 (11)	0.038 (6)*	
H5	0.633 (3)	0.417 (2)	0.6294 (11)	0.033 (5)*	
H7	0.733 (2)	0.2637 (19)	0.5632 (10)	0.028 (5)*	
H8A	0.895 (3)	0.165 (2)	0.4615 (12)	0.022 (6)*	0.864 (3)
H8B	0.840 (3)	0.069 (2)	0.5207 (12)	0.027 (6)*	0.864 (3)
H8C	0.7997	0.0094	0.4910	0.041*	0.136 (3)
H8D	0.9022	0.1507	0.5039	0.041*	0.136 (3)
H9A	0.887 (3)	−0.053 (3)	0.4044 (13)	0.041 (7)*	0.864 (3)
H9B	0.698 (4)	−0.092 (3)	0.4307 (15)	0.045 (7)*	0.864 (3)
H9C	0.8526	0.1963	0.3691	0.023*	0.136 (3)
H9D	0.9350	0.0467	0.3791	0.023*	0.136 (3)
H10A	0.675 (3)	−0.105 (2)	0.2715 (13)	0.032 (6)*	0.864 (3)
H10B	0.547 (3)	−0.133 (2)	0.3318 (14)	0.030 (6)*	0.864 (3)
H10C	0.7177	−0.0255	0.2512	0.027*	0.136 (3)
H10D	0.6670	0.1323	0.2501	0.027*	0.136 (3)
H11A	0.485 (3)	0.063 (2)	0.2104 (12)	0.022 (5)*	0.864 (3)
H11B	0.371 (3)	−0.063 (2)	0.2180 (12)	0.024 (5)*	0.864 (3)
H11C	0.4066	0.0228	0.1928	0.033*	0.136 (3)
H11D	0.4251	−0.0937	0.2552	0.033*	0.136 (3)
H12A	0.818 (3)	0.216 (2)	0.3439 (13)	0.036 (6)*	0.864 (3)
H12B	0.696 (3)	0.181 (2)	0.2665 (13)	0.029 (6)*	0.864 (3)
H12C	0.868 (3)	0.089 (2)	0.2898 (13)	0.037 (7)*	0.864 (3)
H12D	0.6808	−0.1020	0.4341	0.039*	0.136 (3)
H12E	0.7519	−0.1583	0.3620	0.039*	0.136 (3)
H12F	0.5491	−0.1428	0.3616	0.039*	0.136 (3)

### {2-[({2-[(2-Aminoethyl-κN)(methyl)amino-κN]ethyl}imino-κN)methyl]benzenethiolato-κS}nickel(II) chloride (1) Atomic displacement parameters (Å^2^)

**Table:**

	*U*^11^	*U*^22^	*U*^33^	*U*^12^	*U*^13^	*U*^23^
Ni1	0.01721 (11)	0.01974 (11)	0.01704 (11)	0.00138 (8)	0.00044 (8)	0.00006 (8)
Cl1	0.0282 (2)	0.0263 (2)	0.0242 (2)	−0.00019 (16)	0.00157 (17)	0.00158 (16)
S1	0.01838 (19)	0.0278 (2)	0.0201 (2)	0.00215 (16)	−0.00091 (15)	−0.00525 (17)
N1	0.0179 (7)	0.0315 (8)	0.0199 (7)	0.0033 (6)	−0.0013 (6)	0.0028 (6)
N2	0.0220 (7)	0.0199 (7)	0.0227 (7)	0.0028 (6)	0.0034 (6)	0.0028 (6)
N3	0.0236 (8)	0.0236 (7)	0.0200 (7)	−0.0003 (6)	0.0018 (6)	−0.0021 (6)
C1	0.0253 (8)	0.0226 (8)	0.0168 (8)	−0.0018 (7)	0.0020 (7)	−0.0004 (6)
C2	0.0265 (9)	0.0310 (9)	0.0288 (10)	0.0024 (7)	0.0033 (8)	−0.0053 (8)
C3	0.0399 (11)	0.0317 (10)	0.0286 (10)	0.0046 (9)	0.0103 (9)	−0.0052 (8)
C4	0.0494 (12)	0.0298 (9)	0.0196 (9)	−0.0044 (9)	0.0048 (8)	−0.0054 (8)
C5	0.0321 (10)	0.0354 (10)	0.0185 (9)	−0.0071 (8)	−0.0019 (8)	−0.0017 (7)
C6	0.0256 (8)	0.0280 (9)	0.0163 (8)	−0.0027 (7)	0.0008 (7)	0.0010 (7)
C7	0.0229 (8)	0.0372 (10)	0.0186 (8)	−0.0014 (8)	−0.0041 (7)	0.0007 (7)
C8	0.0221 (9)	0.0531 (13)	0.0259 (10)	0.0116 (9)	−0.0027 (8)	0.0009 (9)
C9A	0.0289 (11)	0.0346 (12)	0.0286 (12)	0.0113 (10)	0.0026 (9)	0.0095 (9)
C9B	0.011 (5)	0.022 (6)	0.025 (6)	0.005 (4)	0.004 (5)	−0.005 (5)
C10A	0.0343 (12)	0.0205 (10)	0.0317 (12)	0.0030 (9)	0.0066 (9)	−0.0043 (9)
C10B	0.020 (6)	0.024 (6)	0.025 (6)	−0.001 (5)	0.009 (5)	0.002 (5)
C11	0.0296 (9)	0.0296 (9)	0.0242 (9)	0.0016 (8)	0.0034 (8)	−0.0069 (8)
C12A	0.0256 (11)	0.0299 (11)	0.0286 (11)	−0.0004 (9)	0.0080 (9)	0.0034 (9)
C12B	0.037 (7)	0.016 (6)	0.025 (7)	−0.009 (5)	0.009 (6)	−0.003 (5)

### {2-[({2-[(2-Aminoethyl-κN)(methyl)amino-κN]ethyl}imino-κN)methyl]benzenethiolato-κS}nickel(II) chloride (1) Geometric parameters (Å, º)

**Table:**

Ni1—N1	1.8587 (14)	C7—H7	0.96 (2)
Ni1—N3	1.9268 (14)	C8—C9A	1.526 (3)
Ni1—N2	1.9345 (13)	C8—C9B	1.601 (12)
Ni1—S1	2.1421 (5)	C8—H8C	0.9900
S1—C1	1.7396 (17)	C8—H8D	0.9900
N1—C7	1.289 (2)	C8—H8A	0.96 (2)
N1—C8	1.499 (2)	C8—H8B	0.94 (2)
N2—C10B	1.425 (12)	C11—C10A	1.513 (3)
N2—C9A	1.473 (2)	C11—C10B	1.604 (12)
N2—C10A	1.488 (2)	C11—H11C	0.9900
N2—C9B	1.496 (12)	C11—H11D	0.9900
N2—C12A	1.507 (2)	C11—H11A	0.97 (2)
N2—C12B	1.566 (12)	C11—H11B	0.96 (2)
N3—C11	1.492 (2)	C9A—H9A	1.02 (2)
N3—HN3A	0.93 (2)	C9A—H9B	1.06 (3)
N3—HN3B	0.841 (19)	C10A—H10A	0.97 (2)
C1—C2	1.404 (2)	C10A—H10B	1.02 (2)
C1—C6	1.411 (2)	C12A—H12A	1.02 (3)
C2—C3	1.380 (3)	C12A—H12B	0.99 (2)
C2—H2	0.953 (19)	C12A—H12C	1.00 (2)
C3—C4	1.388 (3)	C9B—H9C	0.9900
C3—H3	0.89 (2)	C9B—H9D	0.9900
C4—C5	1.365 (3)	C10B—H10C	0.9900
C4—H4	0.94 (2)	C10B—H10D	0.9900
C5—C6	1.417 (2)	C12B—H12D	0.9800
C5—H5	0.93 (2)	C12B—H12E	0.9800
C6—C7	1.435 (2)	C12B—H12F	0.9800
			
N1—Ni1—N3	172.68 (6)	H8C—C8—H8D	109.1
N1—Ni1—N2	86.53 (6)	N1—C8—H8A	109.7 (13)
N3—Ni1—N2	86.16 (6)	C9A—C8—H8A	112.8 (13)
N1—Ni1—S1	97.77 (5)	N1—C8—H8B	107.9 (13)
N3—Ni1—S1	89.53 (5)	C9A—C8—H8B	110.8 (14)
N2—Ni1—S1	175.49 (4)	H8A—C8—H8B	108.6 (18)
C1—S1—Ni1	111.00 (6)	N3—C11—C10A	107.33 (15)
C7—N1—C8	114.95 (14)	N3—C11—C10B	106.1 (5)
C7—N1—Ni1	131.91 (12)	N3—C11—H11C	110.5
C8—N1—Ni1	113.07 (11)	C10B—C11—H11C	110.5
C9A—N2—C10A	112.64 (15)	N3—C11—H11D	110.5
C10B—N2—C9B	117.1 (7)	C10B—C11—H11D	110.5
C9A—N2—C12A	111.16 (15)	H11C—C11—H11D	108.7
C10A—N2—C12A	109.86 (15)	N3—C11—H11A	107.3 (12)
C10B—N2—C12B	109.6 (7)	C10A—C11—H11A	112.3 (12)
C9B—N2—C12B	106.3 (7)	N3—C11—H11B	111.8 (12)
C10B—N2—Ni1	110.0 (5)	C10A—C11—H11B	111.6 (13)
C9A—N2—Ni1	105.56 (11)	H11A—C11—H11B	106.5 (17)
C10A—N2—Ni1	106.80 (12)	N2—C9A—C8	106.59 (16)
C9B—N2—Ni1	107.7 (4)	N2—C9A—H9A	112.3 (13)
C12A—N2—Ni1	110.66 (12)	C8—C9A—H9A	112.1 (14)
C12B—N2—Ni1	105.4 (5)	N2—C9A—H9B	102.4 (15)
C11—N3—Ni1	111.83 (11)	C8—C9A—H9B	115.4 (14)
C11—N3—HN3A	107.1 (13)	H9A—C9A—H9B	108 (2)
Ni1—N3—HN3A	104.2 (14)	N2—C10A—C11	107.20 (15)
C11—N3—HN3B	107.7 (13)	N2—C10A—H10A	110.9 (14)
Ni1—N3—HN3B	110.2 (13)	C11—C10A—H10A	113.2 (14)
HN3A—N3—HN3B	115.9 (19)	N2—C10A—H10B	104.3 (13)
C2—C1—C6	117.68 (15)	C11—C10A—H10B	112.4 (14)
C2—C1—S1	117.14 (13)	H10A—C10A—H10B	108.4 (18)
C6—C1—S1	125.17 (13)	N2—C12A—H12A	108.2 (13)
C3—C2—C1	121.38 (18)	N2—C12A—H12B	109.1 (13)
C3—C2—H2	119.5 (11)	H12A—C12A—H12B	106.5 (19)
C1—C2—H2	119.1 (11)	N2—C12A—H12C	110.2 (14)
C2—C3—C4	121.23 (18)	H12A—C12A—H12C	112.4 (19)
C2—C3—H3	118.2 (13)	H12B—C12A—H12C	110.2 (18)
C4—C3—H3	120.6 (13)	N2—C9B—C8	101.8 (7)
C5—C4—C3	118.43 (17)	N2—C9B—H9C	111.4
C5—C4—H4	120.9 (13)	C8—C9B—H9C	111.4
C3—C4—H4	120.7 (13)	N2—C9B—H9D	111.4
C4—C5—C6	122.11 (17)	C8—C9B—H9D	111.4
C4—C5—H5	124.1 (13)	H9C—C9B—H9D	109.3
C6—C5—H5	113.8 (12)	N2—C10B—C11	105.6 (7)
C1—C6—C5	119.10 (16)	N2—C10B—H10C	110.6
C1—C6—C7	124.70 (15)	C11—C10B—H10C	110.6
C5—C6—C7	116.12 (15)	N2—C10B—H10D	110.6
N1—C7—C6	128.09 (16)	C11—C10B—H10D	110.6
N1—C7—H7	118.1 (11)	H10C—C10B—H10D	108.7
C6—C7—H7	113.8 (11)	N2—C12B—H12D	109.5
N1—C8—C9A	106.96 (15)	N2—C12B—H12E	109.5
N1—C8—C9B	102.9 (4)	H12D—C12B—H12E	109.5
N1—C8—H8C	111.2	N2—C12B—H12F	109.5
C9B—C8—H8C	111.2	H12D—C12B—H12F	109.5
N1—C8—H8D	111.2	H12E—C12B—H12F	109.5
C9B—C8—H8D	111.2		
			
N2—Ni1—N1—C7	168.82 (17)	C12A—N2—C9A—C8	71.6 (2)
S1—Ni1—N1—C7	−12.54 (17)	C12B—N2—C9A—C8	−146.3 (7)
N2—Ni1—N1—C8	−7.83 (13)	Ni1—N2—C9A—C8	−48.46 (18)
S1—Ni1—N1—C8	170.81 (12)	N1—C8—C9A—N2	42.9 (2)
Ni1—S1—C1—C2	177.10 (12)	C9B—C8—C9A—N2	−50.5 (5)
Ni1—S1—C1—C6	−3.70 (17)	C10B—N2—C10A—C11	−55.6 (6)
C6—C1—C2—C3	2.3 (3)	C9A—N2—C10A—C11	162.30 (16)
S1—C1—C2—C3	−178.40 (15)	C9B—N2—C10A—C11	−137.0 (9)
C1—C2—C3—C4	−0.2 (3)	C12A—N2—C10A—C11	−73.2 (2)
C2—C3—C4—C5	−1.8 (3)	C12B—N2—C10A—C11	145.6 (6)
C3—C4—C5—C6	1.6 (3)	Ni1—N2—C10A—C11	46.86 (18)
C2—C1—C6—C5	−2.5 (3)	N3—C11—C10A—N2	−45.4 (2)
S1—C1—C6—C5	178.33 (13)	C10B—C11—C10A—N2	50.7 (6)
C2—C1—C6—C7	174.18 (17)	C10B—N2—C9B—C8	173.6 (7)
S1—C1—C6—C7	−5.0 (3)	C9A—N2—C9B—C8	−47.8 (4)
C4—C5—C6—C1	0.5 (3)	C10A—N2—C9B—C8	−127.0 (6)
C4—C5—C6—C7	−176.40 (18)	C12A—N2—C9B—C8	153.0 (8)
C8—N1—C7—C6	−177.03 (18)	C12B—N2—C9B—C8	−63.5 (8)
Ni1—N1—C7—C6	6.4 (3)	Ni1—N2—C9B—C8	49.1 (6)
C1—C6—C7—N1	5.2 (3)	N1—C8—C9B—N2	−53.5 (6)
C5—C6—C7—N1	−178.09 (18)	C9A—C8—C9B—N2	48.0 (4)
C7—N1—C8—C9A	165.20 (17)	C9A—N2—C10B—C11	124.5 (6)
Ni1—N1—C8—C9A	−17.6 (2)	C10A—N2—C10B—C11	50.5 (5)
C7—N1—C8—C9B	−141.0 (5)	C9B—N2—C10B—C11	−168.6 (6)
Ni1—N1—C8—C9B	36.2 (5)	C12A—N2—C10B—C11	−148.4 (9)
Ni1—N3—C11—C10A	23.43 (19)	C12B—N2—C10B—C11	70.2 (9)
Ni1—N3—C11—C10B	−26.0 (5)	Ni1—N2—C10B—C11	−45.2 (8)
C10B—N2—C9A—C8	141.6 (9)	N3—C11—C10B—N2	45.7 (8)
C10A—N2—C9A—C8	−164.64 (17)	C10A—C11—C10B—N2	−53.3 (5)
C9B—N2—C9A—C8	52.5 (5)		

### {2-[({2-[(2-Aminoethyl-κN)(methyl)amino-κN]ethyl}imino-κN)methyl]benzenethiolato-κS}nickel(II) chloride (1) Hydrogen-bond geometry (Å, º)

**Table:**

*D*—H···*A*	*D*—H	H···*A*	*D*···*A*	*D*—H···*A*
N3—H*N*3*A*···Cl1	0.93 (2)	2.40 (2)	3.2245 (17)	148.7 (18)
N3—H*N*3*B*···Cl1^i^	0.841 (19)	2.41 (2)	3.1948 (17)	156.0 (17)

Symmetry code: (i) −*x*+1/2, *y*−1/2, −*z*+1/2.

### [2-({[2-(Piperazin-1-yl-κ2N1,N4)ethyl]imino-κN}methyl)benzenethiolato-κS]nickel(II) hexafluorophosphate dichloromethane monosolvate (2) Crystal data

**Table:**

[Ni(C_13_H_18_N_3_S)]PF_6_·CH_2_Cl_2_	*Z* = 2
*M**_r_* = 536.97	*F*(000) = 544
Triclinic, *P*1	*D*_x_ = 1.788 Mg m^−^^3^
*a* = 8.725 (3) Å	Mo *K*α radiation, λ = 0.71075 Å
*b* = 10.507 (4) Å	Cell parameters from 3095 reflections
*c* = 11.316 (4) Å	θ = 3.3–27.5°
α = 98.065 (4)°	µ = 1.49 mm^−^^1^
β = 101.274 (6)°	*T* = 153 K
γ = 96.150 (5)°	Prism, orange
*V* = 997.6 (6) Å^3^	0.17 × 0.11 × 0.08 mm

### [2-({[2-(Piperazin-1-yl-κ2N1,N4)ethyl]imino-κN}methyl)benzenethiolato-κS]nickel(II) hexafluorophosphate dichloromethane monosolvate (2) Data collection

**Table:**

Rigaku AFC11 with Saturn 724+ CCD diffractometer	4392 independent reflections
Radiation source: Rotating Anode	3240 reflections with *I* > 2σ(*I*)
Detector resolution: 28.5714 pixels mm^-1^	*R*_int_ = 0.041
ω scans	θ_max_ = 27.5°, θ_min_ = 3.3°
Absorption correction: multi-scan (*REQAB*; Rigaku, 1998)	*h* = −10→11
*T*_min_ = 0.828, *T*_max_ = 1.000	*k* = −13→13
8155 measured reflections	*l* = −14→11

### [2-({[2-(Piperazin-1-yl-κ2N1,N4)ethyl]imino-κN}methyl)benzenethiolato-κS]nickel(II) hexafluorophosphate dichloromethane monosolvate (2) Refinement

**Table:**

Refinement on *F*^2^	Primary atom site location: structure-invariant direct methods
Least-squares matrix: full	Hydrogen site location: difference Fourier map
*R*[*F*^2^ > 2σ(*F*^2^)] = 0.037	All H-atom parameters refined
*wR*(*F*^2^) = 0.087	*w* = 1/[σ^2^(*F*_o_^2^) + (0.0389*P*)^2^] where *P* = (*F*_o_^2^ + 2*F*_c_^2^)/3
*S* = 0.93	(Δ/σ)_max_ = 0.001
4392 reflections	Δρ_max_ = 0.79 e Å^−^^3^
333 parameters	Δρ_min_ = −0.52 e Å^−^^3^
0 restraints	

### [2-({[2-(Piperazin-1-yl-κ2N1,N4)ethyl]imino-κN}methyl)benzenethiolato-κS]nickel(II) hexafluorophosphate dichloromethane monosolvate (2) Special details

**Table:**

Geometry. All esds (except the esd in the dihedral angle between two l.s. planes) are estimated using the full covariance matrix. The cell esds are taken into account individually in the estimation of esds in distances, angles and torsion angles; correlations between esds in cell parameters are only used when they are defined by crystal symmetry. An approximate (isotropic) treatment of cell esds is used for estimating esds involving l.s. planes.

### [2-({[2-(Piperazin-1-yl-κ2N1,N4)ethyl]imino-κN}methyl)benzenethiolato-κS]nickel(II) hexafluorophosphate dichloromethane monosolvate (2) Fractional atomic coordinates and isotropic or equivalent isotropic displacement parameters (Å^2^)

**Table:**

	*x*	*y*	*z*	*U*_iso_*/*U*_eq_	
Ni1	0.57594 (4)	0.27835 (3)	0.57343 (3)	0.01844 (11)	
Cl1	0.25537 (9)	0.20611 (8)	0.80312 (7)	0.0387 (2)	
Cl2	0.11870 (9)	0.29079 (8)	1.01271 (7)	0.03346 (19)	
S1	0.32922 (8)	0.21518 (7)	0.51052 (6)	0.02276 (16)	
P1	0.72668 (8)	0.35508 (8)	0.17761 (7)	0.02436 (18)	
F1	0.5860 (3)	0.2992 (3)	0.2304 (3)	0.0855 (9)	
F2	0.6694 (3)	0.2481 (2)	0.0598 (2)	0.0743 (8)	
F3	0.8640 (3)	0.4110 (3)	0.1214 (3)	0.0879 (9)	
F4	0.7800 (3)	0.4640 (3)	0.2946 (2)	0.0931 (10)	
F5	0.8362 (2)	0.2621 (2)	0.24263 (19)	0.0551 (6)	
F6	0.6156 (2)	0.44975 (18)	0.11403 (16)	0.0351 (4)	
N1	0.6501 (2)	0.1584 (2)	0.4715 (2)	0.0199 (5)	
N2	0.7907 (2)	0.3602 (2)	0.6333 (2)	0.0213 (5)	
N3	0.5633 (3)	0.4116 (2)	0.7042 (2)	0.0241 (5)	
C1	0.2997 (3)	0.1243 (3)	0.3646 (3)	0.0203 (6)	
C2	0.1451 (3)	0.1044 (3)	0.2930 (3)	0.0279 (7)	
C3	0.1064 (4)	0.0296 (3)	0.1791 (3)	0.0323 (7)	
C4	0.2205 (4)	−0.0298 (3)	0.1305 (3)	0.0336 (8)	
C5	0.3726 (4)	−0.0106 (3)	0.1968 (3)	0.0286 (7)	
C6	0.4164 (3)	0.0657 (3)	0.3139 (3)	0.0224 (6)	
C7	0.5804 (3)	0.0807 (3)	0.3730 (3)	0.0222 (6)	
C8	0.8213 (3)	0.1579 (3)	0.5183 (3)	0.0255 (6)	
C9	0.8952 (3)	0.2982 (3)	0.5619 (3)	0.0261 (7)	
C10	0.8201 (4)	0.3475 (3)	0.7658 (3)	0.0297 (7)	
C11	0.6707 (4)	0.3764 (3)	0.8096 (3)	0.0302 (7)	
C12	0.7813 (4)	0.4991 (3)	0.6233 (3)	0.0259 (6)	
C13	0.6363 (4)	0.5334 (3)	0.6723 (3)	0.0272 (7)	
C14	0.2908 (4)	0.2570 (4)	0.9634 (3)	0.0385 (8)	
H2	0.070 (3)	0.152 (3)	0.325 (3)	0.034 (9)*	
H3	0.002 (3)	0.016 (3)	0.134 (3)	0.027 (8)*	
H3N	0.479 (3)	0.421 (3)	0.719 (3)	0.019 (8)*	
H4	0.194 (3)	−0.085 (3)	0.047 (3)	0.037 (9)*	
H5	0.451 (3)	−0.054 (3)	0.168 (3)	0.032 (9)*	
H7	0.639 (3)	0.028 (3)	0.332 (3)	0.028 (8)*	
H8A	0.829 (3)	0.111 (3)	0.588 (3)	0.028 (8)*	
H8B	0.869 (3)	0.118 (3)	0.452 (3)	0.027 (8)*	
H9A	1.006 (3)	0.303 (3)	0.611 (3)	0.025 (8)*	
H9B	0.902 (3)	0.344 (3)	0.496 (3)	0.024 (8)*	
H10A	0.841 (3)	0.260 (3)	0.770 (2)	0.015 (7)*	
H10B	0.913 (3)	0.404 (3)	0.812 (3)	0.029 (8)*	
H11A	0.621 (3)	0.303 (3)	0.834 (2)	0.010 (6)*	
H11B	0.687 (3)	0.448 (3)	0.878 (3)	0.033 (9)*	
H12A	0.877 (3)	0.559 (3)	0.667 (3)	0.025 (8)*	
H12B	0.775 (3)	0.514 (3)	0.539 (3)	0.025 (8)*	
H13A	0.667 (3)	0.602 (3)	0.743 (3)	0.020 (7)*	
H13B	0.565 (3)	0.566 (3)	0.616 (3)	0.019 (7)*	
H14A	0.324 (4)	0.184 (4)	1.000 (3)	0.052 (11)*	
H14B	0.365 (4)	0.337 (4)	0.982 (4)	0.064 (13)*	

### [2-({[2-(Piperazin-1-yl-κ2N1,N4)ethyl]imino-κN}methyl)benzenethiolato-κS]nickel(II) hexafluorophosphate dichloromethane monosolvate (2) Atomic displacement parameters (Å^2^)

**Table:**

	*U*^11^	*U*^22^	*U*^33^	*U*^12^	*U*^13^	*U*^23^
Ni1	0.01714 (19)	0.01885 (19)	0.01872 (19)	0.00118 (13)	0.00293 (14)	0.00337 (14)
Cl1	0.0409 (5)	0.0399 (5)	0.0342 (4)	0.0054 (4)	0.0098 (4)	−0.0002 (4)
Cl2	0.0305 (4)	0.0396 (5)	0.0276 (4)	−0.0010 (3)	0.0044 (3)	0.0036 (3)
S1	0.0184 (3)	0.0259 (4)	0.0240 (4)	0.0026 (3)	0.0050 (3)	0.0039 (3)
P1	0.0214 (4)	0.0288 (4)	0.0229 (4)	0.0044 (3)	0.0037 (3)	0.0049 (3)
F1	0.0698 (15)	0.098 (2)	0.136 (2)	0.0414 (15)	0.0698 (17)	0.0879 (19)
F2	0.1012 (18)	0.0459 (15)	0.0566 (15)	0.0286 (13)	−0.0211 (13)	−0.0173 (12)
F3	0.0450 (13)	0.111 (2)	0.137 (3)	0.0190 (14)	0.0501 (16)	0.069 (2)
F4	0.134 (2)	0.0682 (18)	0.0475 (15)	0.0435 (17)	−0.0427 (15)	−0.0239 (14)
F5	0.0545 (13)	0.0671 (16)	0.0495 (13)	0.0363 (11)	0.0028 (10)	0.0201 (12)
F6	0.0394 (10)	0.0343 (11)	0.0340 (10)	0.0125 (8)	0.0044 (8)	0.0123 (8)
N1	0.0178 (11)	0.0194 (12)	0.0234 (12)	0.0029 (9)	0.0028 (10)	0.0086 (10)
N2	0.0187 (12)	0.0214 (12)	0.0224 (12)	−0.0008 (9)	0.0001 (10)	0.0073 (10)
N3	0.0237 (14)	0.0268 (14)	0.0212 (12)	0.0011 (11)	0.0052 (11)	0.0034 (10)
C1	0.0198 (14)	0.0174 (14)	0.0248 (15)	0.0019 (11)	0.0041 (12)	0.0087 (11)
C2	0.0237 (16)	0.0267 (16)	0.0320 (17)	0.0016 (13)	0.0006 (14)	0.0098 (13)
C3	0.0281 (17)	0.0295 (18)	0.0336 (18)	−0.0037 (13)	−0.0073 (15)	0.0117 (14)
C4	0.045 (2)	0.0236 (16)	0.0254 (16)	−0.0033 (14)	−0.0047 (15)	0.0028 (13)
C5	0.0341 (18)	0.0218 (15)	0.0278 (16)	0.0025 (13)	0.0032 (14)	0.0026 (13)
C6	0.0242 (15)	0.0171 (14)	0.0252 (15)	0.0000 (11)	0.0034 (12)	0.0063 (12)
C7	0.0258 (16)	0.0170 (14)	0.0275 (15)	0.0060 (12)	0.0105 (13)	0.0066 (12)
C8	0.0183 (15)	0.0273 (16)	0.0319 (17)	0.0066 (12)	0.0037 (13)	0.0079 (14)
C9	0.0184 (15)	0.0281 (17)	0.0325 (17)	0.0015 (12)	0.0047 (13)	0.0094 (14)
C10	0.0295 (17)	0.0324 (18)	0.0230 (16)	−0.0022 (14)	−0.0059 (13)	0.0115 (14)
C11	0.0401 (19)	0.0314 (18)	0.0177 (15)	0.0002 (14)	0.0036 (14)	0.0069 (13)
C12	0.0256 (16)	0.0237 (16)	0.0271 (16)	−0.0027 (12)	0.0036 (13)	0.0078 (13)
C13	0.0350 (18)	0.0225 (16)	0.0221 (15)	0.0021 (13)	0.0018 (14)	0.0042 (13)
C14	0.0293 (18)	0.051 (2)	0.0314 (19)	0.0023 (17)	−0.0002 (15)	0.0072 (17)

### [2-({[2-(Piperazin-1-yl-κ2N1,N4)ethyl]imino-κN}methyl)benzenethiolato-κS]nickel(II) hexafluorophosphate dichloromethane monosolvate (2) Geometric parameters (Å, º)

**Table:**

Ni1—N1	1.843 (2)	C3—C4	1.391 (5)
Ni1—N3	1.917 (3)	C3—H3	0.94 (3)
Ni1—N2	1.924 (2)	C4—C5	1.369 (4)
Ni1—S1	2.1316 (10)	C4—H4	1.01 (3)
Cl1—C14	1.773 (4)	C5—C6	1.408 (4)
Cl2—C14	1.755 (4)	C5—H5	0.95 (3)
S1—C1	1.745 (3)	C6—C7	1.438 (4)
P1—F3	1.561 (2)	C7—H7	0.93 (3)
P1—F1	1.562 (2)	C8—C9	1.517 (4)
P1—F2	1.578 (2)	C8—H8A	0.98 (3)
P1—F4	1.581 (2)	C8—H8B	0.99 (3)
P1—F5	1.592 (2)	C9—H9A	1.01 (3)
P1—F6	1.6064 (19)	C9—H9B	0.95 (3)
N1—C7	1.285 (3)	C10—C11	1.528 (5)
N1—C8	1.485 (3)	C10—H10A	0.96 (3)
N2—C9	1.476 (4)	C10—H10B	0.96 (3)
N2—C12	1.489 (4)	C11—H11A	0.94 (3)
N2—C10	1.499 (4)	C11—H11B	0.98 (3)
N3—C11	1.482 (4)	C12—C13	1.535 (4)
N3—C13	1.490 (4)	C12—H12A	0.98 (3)
N3—H3N	0.80 (3)	C12—H12B	0.98 (3)
C1—C2	1.409 (4)	C13—H13A	0.97 (3)
C1—C6	1.417 (4)	C13—H13B	0.93 (3)
C2—C3	1.371 (4)	C14—H14A	0.96 (4)
C2—H2	0.96 (3)	C14—H14B	0.97 (4)
			
N1—Ni1—N3	162.95 (10)	C4—C5—H5	120.6 (18)
N1—Ni1—N2	87.80 (10)	C6—C5—H5	117.3 (18)
N3—Ni1—N2	76.05 (10)	C5—C6—C1	119.0 (3)
N1—Ni1—S1	98.90 (7)	C5—C6—C7	116.6 (3)
N3—Ni1—S1	97.67 (8)	C1—C6—C7	124.4 (3)
N2—Ni1—S1	171.77 (7)	N1—C7—C6	127.1 (3)
C1—S1—Ni1	107.93 (9)	N1—C7—H7	119.2 (18)
F3—P1—F1	178.42 (16)	C6—C7—H7	113.7 (18)
F3—P1—F2	89.42 (17)	N1—C8—C9	107.1 (2)
F1—P1—F2	89.56 (17)	N1—C8—H8A	105.7 (16)
F3—P1—F4	91.08 (18)	C9—C8—H8A	110.2 (18)
F1—P1—F4	89.91 (18)	N1—C8—H8B	109.8 (16)
F2—P1—F4	178.47 (14)	C9—C8—H8B	110.4 (17)
F3—P1—F5	90.76 (14)	H8A—C8—H8B	113 (2)
F1—P1—F5	90.47 (13)	N2—C9—C8	106.7 (2)
F2—P1—F5	91.00 (13)	N2—C9—H9A	112.5 (16)
F4—P1—F5	90.44 (13)	C8—C9—H9A	110.4 (17)
F3—P1—F6	89.75 (13)	N2—C9—H9B	107.6 (17)
F1—P1—F6	89.03 (12)	C8—C9—H9B	112.4 (18)
F2—P1—F6	89.73 (12)	H9A—C9—H9B	107 (2)
F4—P1—F6	88.82 (12)	N2—C10—C11	106.3 (2)
F5—P1—F6	179.11 (11)	N2—C10—H10A	106.5 (16)
C7—N1—C8	118.7 (2)	C11—C10—H10A	112.9 (16)
C7—N1—Ni1	131.57 (19)	N2—C10—H10B	111.7 (18)
C8—N1—Ni1	109.78 (18)	C11—C10—H10B	112.7 (17)
C9—N2—C12	114.7 (2)	H10A—C10—H10B	107 (2)
C9—N2—C10	115.9 (2)	N3—C11—C10	106.3 (2)
C12—N2—C10	108.4 (2)	N3—C11—H11A	109.2 (16)
C9—N2—Ni1	110.28 (17)	C10—C11—H11A	111.3 (16)
C12—N2—Ni1	103.48 (17)	N3—C11—H11B	107.8 (19)
C10—N2—Ni1	102.70 (17)	C10—C11—H11B	114.8 (17)
C11—N3—C13	109.2 (2)	H11A—C11—H11B	107 (2)
C11—N3—Ni1	102.14 (19)	N2—C12—C13	106.3 (2)
C13—N3—Ni1	104.61 (18)	N2—C12—H12A	114.1 (17)
C11—N3—H3N	111 (2)	C13—C12—H12A	111.2 (16)
C13—N3—H3N	110 (2)	N2—C12—H12B	111.3 (18)
Ni1—N3—H3N	119 (2)	C13—C12—H12B	112.4 (17)
C2—C1—C6	117.5 (3)	H12A—C12—H12B	102 (2)
C2—C1—S1	116.8 (2)	N3—C13—C12	106.1 (3)
C6—C1—S1	125.6 (2)	N3—C13—H13A	111.2 (17)
C3—C2—C1	121.9 (3)	C12—C13—H13A	110.8 (16)
C3—C2—H2	121.7 (18)	N3—C13—H13B	111.6 (17)
C1—C2—H2	116.2 (18)	C12—C13—H13B	112.1 (17)
C2—C3—C4	120.4 (3)	H13A—C13—H13B	105 (2)
C2—C3—H3	120.1 (17)	Cl2—C14—Cl1	112.25 (19)
C4—C3—H3	119.5 (17)	Cl2—C14—H14A	106 (2)
C5—C4—C3	119.1 (3)	Cl1—C14—H14A	106 (2)
C5—C4—H4	119.4 (17)	Cl2—C14—H14B	108 (2)
C3—C4—H4	121.5 (17)	Cl1—C14—H14B	107 (2)
C4—C5—C6	121.9 (3)	H14A—C14—H14B	117 (3)
			
N3—Ni1—N1—C7	−178.7 (3)	C5—C6—C7—N1	170.2 (3)
N2—Ni1—N1—C7	−160.0 (3)	C1—C6—C7—N1	−8.5 (5)
S1—Ni1—N1—C7	15.1 (3)	C7—N1—C8—C9	138.9 (3)
N3—Ni1—N1—C8	1.5 (5)	Ni1—N1—C8—C9	−41.2 (3)
N2—Ni1—N1—C8	20.09 (19)	C12—N2—C9—C8	−146.0 (2)
S1—Ni1—N1—C8	−164.72 (18)	C10—N2—C9—C8	86.4 (3)
Ni1—S1—C1—C2	−162.6 (2)	Ni1—N2—C9—C8	−29.6 (3)
Ni1—S1—C1—C6	19.5 (3)	N1—C8—C9—N2	45.1 (3)
C6—C1—C2—C3	1.1 (4)	C9—N2—C10—C11	−161.6 (2)
S1—C1—C2—C3	−177.0 (2)	C12—N2—C10—C11	67.7 (3)
C1—C2—C3—C4	0.1 (5)	Ni1—N2—C10—C11	−41.3 (3)
C2—C3—C4—C5	−1.3 (5)	C13—N3—C11—C10	−64.2 (3)
C3—C4—C5—C6	1.1 (5)	Ni1—N3—C11—C10	46.2 (3)
C4—C5—C6—C1	0.1 (5)	N2—C10—C11—N3	−2.9 (3)
C4—C5—C6—C7	−178.7 (3)	C9—N2—C12—C13	164.6 (2)
C2—C1—C6—C5	−1.2 (4)	C10—N2—C12—C13	−64.2 (3)
S1—C1—C6—C5	176.7 (2)	Ni1—N2—C12—C13	44.4 (3)
C2—C1—C6—C7	177.5 (3)	C11—N3—C13—C12	67.6 (3)
S1—C1—C6—C7	−4.7 (4)	Ni1—N3—C13—C12	−41.1 (3)
C8—N1—C7—C6	179.4 (3)	N2—C12—C13—N3	−2.2 (3)
Ni1—N1—C7—C6	−0.4 (5)		

### [2-({[2-(Piperazin-1-yl-κ2N1,N4)ethyl]imino-κN}methyl)benzenethiolato-κS]nickel(II) hexafluorophosphate dichloromethane monosolvate (2) Hydrogen-bond geometry (Å, º)

*Cg*6 is the centroid of the C1–C6 ring.

**Table:**

*D*—H···*A*	*D*—H	H···*A*	*D*···*A*	*D*—H···*A*
N3—H3*N*···F6^i^	0.80 (3)	2.50 (3)	3.114 (3)	135 (3)
C10—H10*A*···C4^ii^	0.96 (3)	2.85 (3)	0.0000 (5)	147 (2)
C8—H8*A*···*Cg*6^ii^	0.98 (3)	2.84 (3)	3.778 (4)	160 (2)

Symmetry codes: (i) −*x*+1, −*y*+1, −*z*+1; (ii) −*x*+1, −*y*, −*z*+1.
